# Fracture Strength of Composite Rest Seats: An In Vitro Comparative Study of Different Composite versus Amalgam Restorations

**DOI:** 10.3390/ma16134830

**Published:** 2023-07-05

**Authors:** Shaimaa M. Fouda, Passent Ellakany, Mohammed M. Gad, Hala A. Bahgat, Neveen M. Ayad, Ijlal Shahrukh Ateeq, Laila Al Dehailan, Amr A. Mahrous

**Affiliations:** 1 Department of Substitutive Dental Sciences, College of Dentistry, Imam Abdulrahman Bin Faisal University, P.O. Box 1982, Dammam 31441, Saudi Arabia; 2 Department of Removable Prosthodontics, College of Dentistry, Al-Ahram Canadian University, 6th of October 3221405, Egypt; 3 Department of Restorative Dental Sciences, College of Dentistry, Imam Abdulrahman Bin Faisal University, P.O. Box 1982, Dammam 31441, Saudi Arabialdehailan@iau.edu.sa (L.A.D.); 4 Biomedical Engineering Department, College of Engineering, Imam Abdulrahman Bin Faisal University, P.O. Box 1982, Dammam 31441, Saudi Arabia

**Keywords:** dental materials, composite dental resins, fracture strength, removable partial denture

## Abstract

Occlusal rest provides support for removable partial dentures (RPD). Rest seats are ideally prepared in enamel, but the abutment teeth might be restored or need restorations. This study compared the fracture strength of abutments restored with composite to amalgam restorations after rest seat preparation. Disto-occlusal cavities were prepared in 30 extracted human maxillary premolars. The specimens were allocated in three groups (n = 10) based on the type of restoration. All the specimens were exposed to thermomechanical aging followed by cycling loading. Fracture strength was tested using a universal testing machine, and then, the fracture mode was recorded. The data were analyzed using Kruskal–Wallis test with a significance level set at 0.05. The fracture mode was recorded as catastrophic or non-catastrophic. The fracture strength between all tested groups showed no significant difference. The highest and lowest fracture strength were recorded on amalgam and Tetric N-Ceram groups, respectively. Composite Tetric N-Ceram showed equal distribution of fracture sites on the restorative materials and teeth, it also displayed the highest number of non-catastrophic fractures unlike other groups where the fracture occurred more within the restorations. The fracture strength of composite was comparable to that of amalgam restorations with prepared rest seats.

## 1. Introduction

Removable partial dentures (RPD) could be an optimum choice in treating partially edentulous patients. The main advantages of RPD are cost effectiveness, conservative tooth preparation, and ease of fabrication [1,2]. Occlusal rest is a component of the RPD that provides vertical support. It allows the dissipation of forces on the abutment teeth in an axial direction, maintains the prosthesis in the planned location and prevents impingement on the soft tissues [1,2,3]. 

Rest seats are ideally prepared on the enamel of abutment teeth. They could also be included in indirect cast restorations, surveyed crowns, or amalgam restorations [2,3]. Computer aided designing and computer aided manufacturing technology (CAD-CAM) is increasingly used in the fabrication of dental restorations. Valenti et al. [4] reported comparable mechanical properties between milled and 3D-printed dental restorations, although milled restorations showed better flexural strength after aging than 3D-printed restorations. Future studies are required to investigate the effect of adding rest seat preparations on the mechanical properties of CAD-CAM restorations fabricated by different technologies. 

Amalgam restorations are recommended in restoring posterior teeth that are subjected to heavy masticatory forces, because of their high strength, low wear, excellent marginal adaptability, and affordable price [5]. However, the unfavorable creep and corrosion properties of amalgam could cause restoration failure [6].

Recently, resin-based composites were used extensively in restoring posterior teeth. They provide tooth-like appearance in addition to overcoming the drawbacks of amalgam restorations [6]. Also, composites can replace ceramics in restoring damaged teeth. Composite tooth preparation is more conservative than ceramics as well as having an affordable cost, limited chair side time, ease of placement, and repair intraorally [7]. 

The mechanical properties of dental restorations such as modulus of elasticity, flexural strength, surface roughness and fracture toughness are determinants of the restoration longevity [8]. Occlusal forces exerted by the patients having RPDs range between 65 and 235 Newton [2]. Moreover, during function, dental restorations are subjected to cyclic loading and aging processes that incorporate exposure to several fluids at different temperatures [8,9]. Accordingly, dental restorations should have sufficient strength to withstand the loading force and aging effects. 

Recently, several types of composite resins have been introduced including variable filler sizes and contents with improved mechanical properties [10]. These composite resins could possibly receive an occlusal rest seat. However, studies that have investigated the behavior of composite restorations supporting occlusal rests are very limited. The purpose of this study was to evaluate and compare the fracture strength of two different types of composite resins to amalgam with prepared occlusal rest seats in simulated oral conditions. The study hypothesis states that the fracture strength of composite and amalgam restorations having a prepared occlusal rest seat would be comparable.

## 2. Materials and Methods

### 2.1. Study Design

The sample size was calculated using the World Health Organization formula with 0.05 level of significance and 80% power, and revealed the need for 10 specimens/group. The study was approved by the Institutional Review Board, at Imam Abdulrahman Bin Faisal University, Dammam, Saudi Arabia (IRB 2023-02-173). Thirty sound permanent maxillary first premolars extracted for orthodontic or periodontal reasons and of similar crown dimensions were collected. All teeth were cleaned by immersion in 5.25% sodium hypochlorite for 1 day then stored in distilled water at room temperature for 1 day before testing [11]. 

The teeth were seated vertically in an auto-polymerized acrylic resin blocks where the cement enamel junction (CEJ) of the crown was 1 mm apical to the resin block.

**Figure 1:**
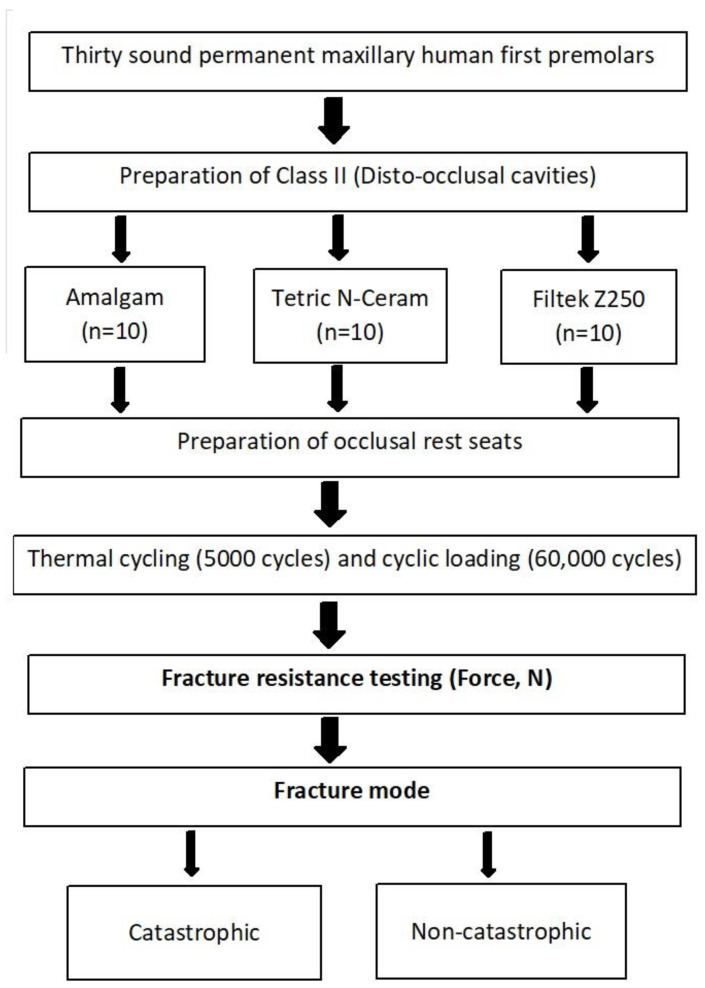
Study design and workflow.

The study design and workflow are illustrated in Figure 1. 

### 2.2. Preparation of Class II (Disto-Occlusal Cavities)

**Figure 2:**
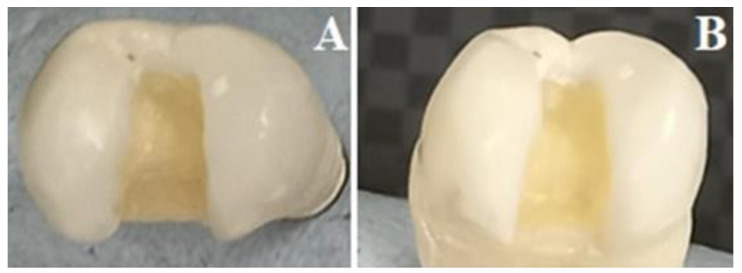
Prepared Class II disto-occlusal cavity. (**A**) Occlusal view, (**B**) Proximal view.

Class II disto-occlusal cavities (Figure 2) were prepared in all teeth by single operator [A.M.] using a high-speed handpiece with a water coolant. The proximal box width was 1/3 the bucco-lingual distance, axial wall was extended 1.5 mm deep, and the gingival margin was located 1 mm occlusal to the CEJ. The occlusal segment of the isthmus width was prepared not exceeding 2/3 of the inter-cuspal distance, 1.5 mm depth, and parallel facial and lingual walls. The cavo-surface margins were 90°, with rounded internal line and point angles. Caliper and periodontal probes were used to ensure that all preparations were of standardized dimensions [11,12]. 

### 2.3. Restorative Procedures of Class II Cavities and Grouping of the Specimens

The prepared teeth with Class II cavities were randomly allocated in 3 groups (n = 10) according to the type of the restorative materials used (Table 1). 

All the specimens were restored using Tofflemire bands (no. 101, thickness: 0.04 mm) in the retainer (TofflemireRetainer Universal 1140, KerrHawe, Bioggio, Switzerland or AutoMatrix, Dentsply, Konstanz, Germany). The first group was restored with high copper amalgam alloy following the manufacturer’s guidelines. Amalgam was triturated then condensed manually using a plugger under maximum condensation stress. Amalgam carving was carried out after cavity overfilling followed by surface burnishing. After complete setting of the amalgam, the matrix band was removed, and final finishing was performed [11,12]. The second and third groups were restored with micro-hybrid, and nanohybrid composite resins, respectively (shade A3). Etching procedure was performed by the application of 37% phosphoric acid gel, rinsing with water and drying then followed by application of the relevant dental adhesive for each composite resin and curing for 20 s at 7 mm away from occlusal, mesial, and distal directions by Light emitting diode (L.E. Demetron I, Kerr Corporation, Orange, CA, USA) of 790 mW/cm^2^) [13]. This was carried out to reduce the shrinkage stresses that might occur during the polymerization of the adhesive. The composites were added in an incremental layering technique and irradiated for 40 s according to the manufacturers’ guidelines using the same Light emitting diode unit. The matrix band was then removed, and final finishing was carried out with aluminum oxide discs (Sof-Lex Disks, 3M, St. Paul, MN, USA) of increasing grit order (360 and 600) under proper water-cooling system [14]. 

### 2.4. Preparation of Occlusal Rest Seats

The restored teeth were placed in distilled water at 37 °C for 1 day to achieve complete setting of the restorative materials. One investigator (S.M.F.) prepared the rest seats in the form of a rounded triangle with a saucer or spoon shaped floor (2 × 2.5 × 1.5 mm). 

### 2.5. Thermocycling Procedure

The teeth were then exposed to 5000 thermomechanical aging cycles at temperatures between 5 °C and 55 °C in thermal cycling machine (Thermocycler THE-1100—SD Mechatronik GmbH, Feldkirchen-Westerham, Germany) with 30 s soaks in a water bath at each respective temperature, and 30 s transfer between the temperature baths, simulating short-term aging for 6 months [15]. 

Then, the specimens underwent cyclic loading at 50 Newtons (N) using a chewing simulator (CS-4.2, SD Mechatronik GMBH, Feldkirchen, Germany). The load cycle was set to have 2 mm vertical descending movement for 60,000 cycles, which is equivalent to 3 months of clinical service [16]. 

### 2.6. Fracture Strength Testing

The fracture strength was evaluated using a universal testing machine (Instron 8871 Universal Testing Machine, Instron, Shakopee, MN, USA), with a customized steel rounded-end indenter (2.5-mm radius) to apply the compressive load with a crosshead speed at 0.5 mm/min. The specimens were subjected to a vertical load centralized on the rest seat, until fracture. Maximum load till fracture was documented in N for each specimen.

After testing, teeth segments were examined by three different investigators under optical microscope (Nikon, H550L, Tokyo, Japan) to record the fracture mode. Fractures were classified according to the extent fracture as follows: catastrophic when the extent of fracture passed the CEJ apically and non-catastrophic when the fracture was occlusal to CEJ [17]. 

### 2.7. Statistical Analysis

The mean and standard deviation of each group were calculated. The statistical analysis was performed using Statistical Package for Social Sciences (SPSS/version 21) software. A normality test conducted using Shapiro–Wilk test showed that not all the data under study were even approximately normal and the research variables were not normally distributed. Therefore, non-parametric test Kruskal–Wallis test (K-test) was used for the overall significance within the group. The level of significance was set at 0.05.

## 3. Results 

### 3.1. Fracture Resistance

**Figure 3:**
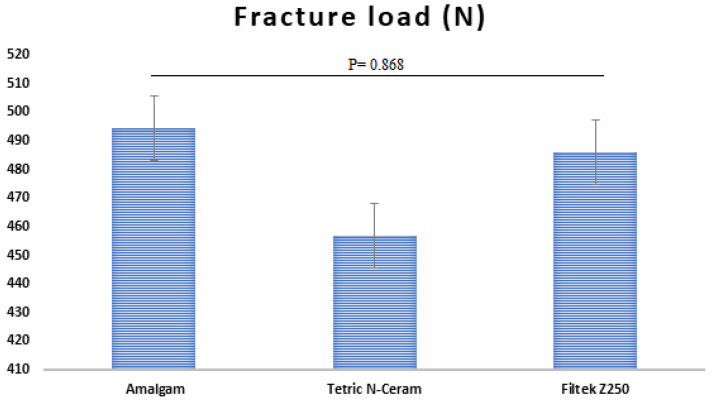
Mean Fracture Load and significant between the tested restorative materials.

Means, standard deviations (SD), and significances are presented in Figure 3. Kruskal–Wallis test revealed no significances between all tested groups (*p* = 0.868). The highest fracture resistance was recorded for amalgam (494.4 ± 160.8 N) followed by composite Filtek Z250 (486.0 ± 156.3 N), while composite Teric N-Ceram showed the lowest fracture resistance (456.8 ± 123.9 N). 

### 3.2. Fracture Site

Regarding the fracture site, the fracture commonly occurred within the restorative materials except in composite Tetric N-Ceram specimens which showed equal distribution of fracture site in the restorative materials and teeth. Composite Tetric N-Ceram displayed the highest number of non-catastrophic fracture followed by amalgam, while composite Filtek Z250 showed the least fracture (Table 2).

## 4. Discussion

The present study compared the fracture strength of different composite resins to amalgam restorations with prepared occlusal rest seats for RPDs. The study hypothesis was accepted since the fracture strength of the studied restorative materials showed no significant differences. 

Dental amalgam exhibited comparable fracture strength to composite Filtek Z250 and composite Tetric N-Ceram. This finding is in agreement with those of El Okl et al. study [11]. In the current study, spherical uni-compositional high copper amalgam (Megalloy) was used. Spherical alloys require less mercury than typical admixed alloys because of the smaller surface area per volume, and thus, provide better mechanical performance than their counterpart [18].

Farzin et al. [19] found significant lower fatigue strength of amalgam in comparison to composite restorations, with no significant difference between the two composite groups. This discrepancy could be due to the variations in the study design such as the smaller number of cycles for thermal loading (500) performed unlike, in this present study, the different types of dental amalgam (admixed high copper amalgam) and the difference in testing protocol used. In addition, Farzin et al. [19] tested the flexural fatigue resistance of these restorations with prepared rest seat, while we tested their fracture strength. Pospiech et al. [20] found significantly higher fracture resistance of amalgam than composite materials, while no significant differences were reported between the tested composite materials. The variations between the results might be related to the differences in the tested materials and the applied methodology. 

The comparable fracture strength of the investigated composites compared to amalgam confirmed that the performance of composite resins in multi-surface restorations is at par with amalgam, as stated by Palotie et al. [21]. Also, these findings were confirmed by those of Bonilla et al. [22], who stated that composite and amalgam restorative materials have similar fracture toughness values. Incremental packing of composite resin provides proper micromechanical adhesion to enamel and dentin, thus allowing for conservative cavity preparation, as well as strengthening the remaining fragile dental structure [23]. This fact, along with the lower modulus of elasticity of composite resins compared to dental amalgam, may explain the optimum distribution of functional stresses along the restorative material–tooth interface of composite resin resulting in comparable fracture strength to that of dental amalgam. Moreover, a previous finite element analysis study found that composite restorations including rest seat preparation leads to a reduction in the stresses generated inside the tooth, without reducing the tooth ability to withstand the functional occlusal forces [24]. Composite restorations can act as a cushion below the occlusal rests of RPDs absorbing the occlusal forces due to its resiliency [24].

The fracture strength of composite Filtek Z250 and Tetric N-Ceram were similar in this study, this might be due to their structural composition. The greater filler weight fraction of composite Filtek Z250 (84.5%) and its smooth spherical shaped filler particles provide better packing efficiency; hence, the mechanical properties are improved through the stress transfer and the uniform distribution between filler particles in the composite resin [25]. Moreover, the inclusion of pre-polymerized fillers in the composite Tetric N-Ceram might improve the fracture resistance, as suggested by Ramdas et al. [25] and Blackham et al. [26]. Similarly, other studies reported similar results of comparable strength of different types of composite resins [11,19,20]. Furthermore, Mesallum et al. [27] reported a similarity in the fracture behavior of different composite resins supporting occlusal rests of RPDs under functional loading.

The non-catastrophic mode of fracture was more noticeable in composite Tetric-N Ceram, than in composite Filtek Z250. This higher incidence of non-catastrophic fracture mode among composite Tetric N-Ceram might be resulting from the lower polymerization shrinkage stresses generated by the presence of stress relievers in its composition, thus preserving the adhesive bond between tooth surface, adhesive, and restorative material [28,29].

The current study evaluated the fracture strength of two different composite resins in relation to high copper amalgam with prepared rest seats. The specimens were exposed to thermomechanical aging and cyclic loading to simulate the oral conditions of exposure to masticatory load and temperature changes. It is important that fatigue resistance and durability of dental restorations receiving a rest seat are evaluated after artificial aging, masticatory loading, and thermal changes.

The number of thermal cycles selected in this study was 5000, which corresponds to 6 months of clinical situation based on the previous reports that 10,000 thermal cycles are equal to 1 year of clinical usage [15]. The temperature of thermal cycling used varied between 5 °C and 55 °C to simulate the minimum and maximum temperatures of the food and beverages consumed by the patients.

During mastication, the dental restorations are subjected to loading and unloading cycles. These cycles might lead to a reduction of strength and fracture due to fatigue [9]. The occlusal forces exerted by patients having RPDs ranges between 65 and 235 N [2]. Additionally, the maximum biting force applied on a single denture tooth of the RPD is 88 N and the estimated load transferred by the clasp and the rest to the underlying restoration is 49 N [20]. The load applied on the tested specimens in the present study was 50 N for 60,000 cycles corresponding to 3 months of clinical use [16]. The reported fracture strength of the tested materials in this study was between 456.8 and 494.4 N, thus exceeding the highest occlusal forces applied by patients using RPD. 

Comparable fracture strength of composite and amalgam restorations after preparation of rest seat and exposure to artificial aging, suggests the suitability of composite restorations to receive a rest seat preparation. In agreement, a previous longitudinal clinical study found that the rest seats stayed stable and intact after 2 years of clinical use despite the type of restorative material supporting the occlusal rests of RPDs [30].

Future studies are required to assess the effect of in vivo conditions that include salivary secretions, different teeth dimensions, variable temperatures of ingested diet, and fluctuating pH along with exposure to food, beverages, mouth rinses, variety of bacteria, and enzymes. In addition, SEM and cross-sectional microtone images of teeth specimens restored with different restorative materials need to be examined since it would add more information on surface topography of the tested materials. Moreover, the extent of wear and surface roughness encountered in composite resins resulting from friction with occlusal rests of RPDs requires assessment. Future investigations with different composite brands and different fabrication methods including indirect and CAD-CAM fabricated restorations are recommended.

## 5. Conclusions

Fracture strength of amalgam and composite restorations with prepared rest seats showed comparable results. Recent types of composite resins (micro-hybrids or nanohybrids) are acceptable alternatives to high copper amalgam restorations for the preparation of occlusal rest seats supporting RPDs. 

## Figures and Tables

**Table 1:** Restorative materials used in this study.

Commercial Name	Type of Restorative Material	Type of Resin Matrix	Type of Inorganic Fillers	Filler Loading	Manufacturer
Megalloy EZ,	Unicomposition high copper amalgam (non γ_2_)	NA	NA	NA	Dentsply Caulk, Milford, DE, USA
Filtek Z250	Micro-hybrid composite restorative material	Bis-GMA, TEGDMA, UDMA, Bis-EMA,	Combination of non-agglomerated/non-aggregated 20 nm silica filler, Non-agglomerated /non-aggregated zirconia filler, and aggregated zirconia/silica cluster filler 0.01–3.5 µm With an average of 0.6 µm	84.5% by weight 60% by volume	3M ESPE, St. Paul, MN, USA,
TetricN ceram	Nano-hybrid composite restorative material	Bis-GMA, UDMA, TEGDMA, Bis-EMA	Barium glass, ytterbium trifluoride, mixed oxides, silicon dioxide (63.5 wt%) Prepolymerized fillers (17 wt%)	81% by weight 57% by volume	Ivoclar Vivadent AG, Schaan, Liechtenstein

Abbreviations: Bis-GMA = Bis-phenol A di-glycidyl methacrylate, TEGDMA = Tri-ethylene glycol di-methacrylate, UDMA = Urethane di-methyl-methacrylate, Bis-EMA = Bisphenol A di-glycidyl methacrylate ethoxylated.

**Table 2:** Comparison between tested groups regarding the site of fracture and fracture mode.

Fracture Site	Amalgam	Tetric N-Ceram	Filtek Z250
Filling materials	7	5	8
Teeth	3	5	2
**Fracture Mode**	**Amalgam**	**Tetric N-Ceram**	**Filtek Z250**
Catastrophic	0	1	1
non-catastrophic	3	4	1

## Data Availability

The data are available with the corresponding author upon request.
